# The Treatment of Gout

**Published:** 1902-04-05

**Authors:** 


### THE TREATMENT OF GOUT.

Dr. Arthur Luff [1] says that he has no doubt as to the beneficial effects of employing a potassium salt in conjunction with colchicum in the treatment of the acute and subacute gout, and his experience is that the citrate is the most useful. Not only does it tend to diminish the acidity of the urine, and by increasing the solvent power of the urine for the uric acid salts assist their elimination, but it inhibits the conversion of the soluble gelatinous sodium biurate into the comparatively insoluble crystalline form. Lithium salts are not so useful; they have not the same inhibiting effect upon the conversion of the biurate from the gelatinous to the crystalline form, and they have no better solvent effect on gouty deposits. The great objection to them, however, is their greater toxicity and depressing action on the heart as compared with the potassium salts. In the treatment of the gouty state associated with disturbance of the liver the alkaline sodium salts are especially useful. There is no better treatment at the outset than a dose of blue pill or calomel at night, followed by Epsom salts or Carlsbad salts in the morning. In cases of gouty hepatic inadequacy a most useful mixture is one containing sodium bicarbonate, gentian, and nux vomica, taken a quarter of an hour before meals. Massage should never be employed in acute gout, but in cases of subacute or chronic gout, where the joints remain swollen or œdematous and are the seat of considerable deposits, much benefit is frequently derived from massage and galvanism (5 to 10 milliampères to each joint for a few minutes, with the negative pole over the affected joint, followed by a few minutes' massage). Radiant heat and superheated air baths are also useful. They increase the oxidative processes within the body, causing a temporary elevation of the body temperature, marked reddening of the skin of the part treated, and various general changes with considerable amelioration and sometimes complete disappearance of the pain. Radiant heat has a greater penetrating effect than other forms of heat, and is more stimulating. Many cases of chronic gout, however, do not respond to treatment by heat. Dr. Luff's experience is that the whole-body bath is in all cases more useful than merely local applications, with, in the case of the radiant heat bath, an extra localisation of the rays on the affected part. In cases of chronic gout with much deposit in the joints, vapour baths and massage often do more good than heat baths. In the acute or subacute stages of gout, or when a slight attack has just started, the use of the Turkish bath is most undesirable. It is often followed by an exacerbation of the attack or by its extension to joints not at first affected.

[1] Practitioner, March.

